# Structure-Guided Design of Cyclic Peptide: A Potent Inhibitor Targeting PD-1/PD-L1 Axis with Antitumor Activity

**DOI:** 10.3390/ijms262311308

**Published:** 2025-11-22

**Authors:** Wenyu Peng, Wenyu Gu, Wujuan Chen, Jiazheng Zhao, Nuela-Manka’a Che Ajuyo, Yechun Pei, Yi Min, Dayong Wang

**Affiliations:** 1 Key Laboratory of Tropical Resources, School of Pharmaceutical Sciences, Hainan University, The Ministry of Education of the People’s Republic of China, Haikou 570228, China; 2 Laboratory of Biopharmaceuticals and Molecular Pharmacology, One Health Cooperative Innovation Center, Hainan University, Haikou 570228, China; 3 Department of Biotechnology, School of Life and Health, Hainan University, Haikou 570228, China

**Keywords:** PD-1, PD-L1, cyclic peptide, immune checkpoint blockade, protein–protein interaction, cancer immunotherapy

## Abstract

Blocking the protein–protein interaction (PPI) between programmed cell death protein 1 (PD-1) and its ligand PD-L1 is a crucial strategy in cancer immunotherapy. However, existing monoclonal antibody-based therapies have limitations such as high production costs and poor tumor penetration. In this study, we developed a novel cyclic peptide inhibitor, PD-1-0520, through structure-based design. Starting from key fragments of PD-L1 that interact with PD-1, we designed 5 mimetic peptides and further optimized them into 22 cyclic peptide candidates. Through molecular dynamics screening and in vitro and in vivo experimental validation, PD-1-0520 was proven to have potent antitumor activities. Results showed that PD-1-0520 effectively inhibited the PD-1/PD-L1 interaction, restored the immune activity of tumor-infiltrating T cells, and achieved a 68% tumor inhibition rate in B16-F10 tumor-bearing mice without systemic toxicity. It promoted CD8^+^ T cell infiltration into tumors and upregulated activation markers, remodeling the tumor immune microenvironment. These findings demonstrate that PD-1-0520 is a promising immune checkpoint inhibitor, and our design strategy provides a new approach for developing PPI-targeting bioactive inhibitors.

## 1. Introduction

Immune checkpoint blockade has revolutionized cancer treatment, providing significant clinical benefits in multiple tumor types. In particular, monoclonal antibodies targeting the programmed cell death protein 1 (PD-1) and its ligand PD-L1 have demonstrated durable responses and improved overall survival in patients with advanced malignancies including melanoma, non-small cell lung cancer, renal cell carcinoma, and various other solid tumors [1]. The clinical success of these therapies has fundamentally transformed the landscape of oncology, establishing immunotherapy as a fourth pillar of cancer treatment alongside surgery, chemotherapy, and radiation therapy [2].

PD-1 is an inhibitory receptor expressed on activated T cells, B cells, and myeloid cells, with its expression notably increased following T cell activation. Its engagement with PD-L1, which is frequently overexpressed in tumor cells and tumor-infiltrating immune cells, leads to T cell exhaustion, anergy, and immune evasion [3]. This immune checkpoint serves as a physiological “brake” on the immune system to prevent autoimmunity under normal conditions. However, tumors exploit this pathway to escape immune surveillance, creating an immunosuppressive microenvironment that facilitates cancer progression and metastasis.

Despite their remarkable success, antibody-based immune checkpoint inhibitors have notable limitations that potentially restrict their efficacy and broader application. These include high manufacturing costs ($100,000–$200,000 per patient annually), potential immunogenicity, and limited tumor penetration due to their large molecular size (approximately 150 kDa) [4]. Additionally, immune-related adverse events associated with systemic immune activation can lead to severe toxicities in some patients, affecting organs such as the skin, gastrointestinal tract, liver, and endocrine system [5]. Furthermore, only a subset of patients responds to current antibody therapies, highlighting the need for alternative approaches to immune checkpoint blockade.

To overcome these limitations, peptide-based inhibitors have emerged as promising alternatives with distinct advantages. Cyclic peptides, in particular, exhibit improved proteolytic stability, enhanced receptor binding affinity, and better pharmacokinetic properties compared to their linear counterparts [6]. Their smaller size (typically 1–2 kDa) facilitates superior tissue penetration, potentially enabling more effective delivery to the tumor microenvironment [7]. Furthermore, due to their structural flexibility and ability to adopt well-defined conformations, cyclic peptides can effectively mimic key binding motifs of protein–protein interactions (PPIs), enabling them to competitively inhibit critical signaling pathways such as PD-1/PD-L1 [8]. Additionally, peptide-based therapeutics generally demonstrate lower production costs and reduced immunogenicity compared to monoclonal antibodies.

Recent structural studies have elucidated the detailed interface between PD-1 and PD-L1, highlighting specific residues essential for their interaction [9]. Crystallographic analyses have revealed that the binding interface consists of a relatively flat surface dominated by hydrophobic interactions, with key “hot spots” that contribute significantly to binding energy [10]. These findings provide a rational basis for designing peptide inhibitors that mimic PD-L1 structural motifs to block PD-1 binding and restore antitumor immune responses. However, the development of cyclic peptides targeting PD-1/PD-L1 remains in its infancy, and there is a pressing need to explore novel design strategies for potent and selective inhibitors [11].

Previous attempts to develop small molecule and peptide-based PD-1/PD-L1 inhibitors have faced challenges including insufficient binding affinity, poor stability in physiological conditions, and limited in vivo efficacy. The complex nature of the PD-1/PD-L1 interaction interface, which lacks deep binding pockets typically targeted by small molecules, presents a particular challenge for drug design. Cyclic peptides offer a promising solution to this problem, as they can cover larger interface areas while maintaining conformational stability [12].

In this study, we employed a structure-based design approach to develop novel cyclic peptide inhibitors of the PD-1/PD-L1 interaction. Starting from key PD-L1 fragments involved in PD-1 binding, we designed and systematically optimized a series of peptides through computational modeling, molecular dynamics simulations, and experimental validation. Our strategy focused on preserving critical binding residues while enhancing stability and bioavailability through cyclization and strategic amino acid substitutions. This approach led to the identification of PD-1-0520, a cyclic peptide with potent activity against the PD-1/PD-L1 interaction both in vitro and in vivo [13].

The development of effective peptide-based immune checkpoint inhibitors represents a significant advancement in cancer immunotherapy, potentially offering advantages of improved tumor penetration, reduced manufacturing costs, and diminished side effects compared to antibody therapies [14]. Moreover, our structure-guided design strategy provides a valuable framework that could be applied to target other clinically relevant protein–protein interactions in cancer and beyond.

## 2. Results

### 2.1. Identification of Hotspot Residues at the PD-1/PD-L1 Interface

**Figure 1:**
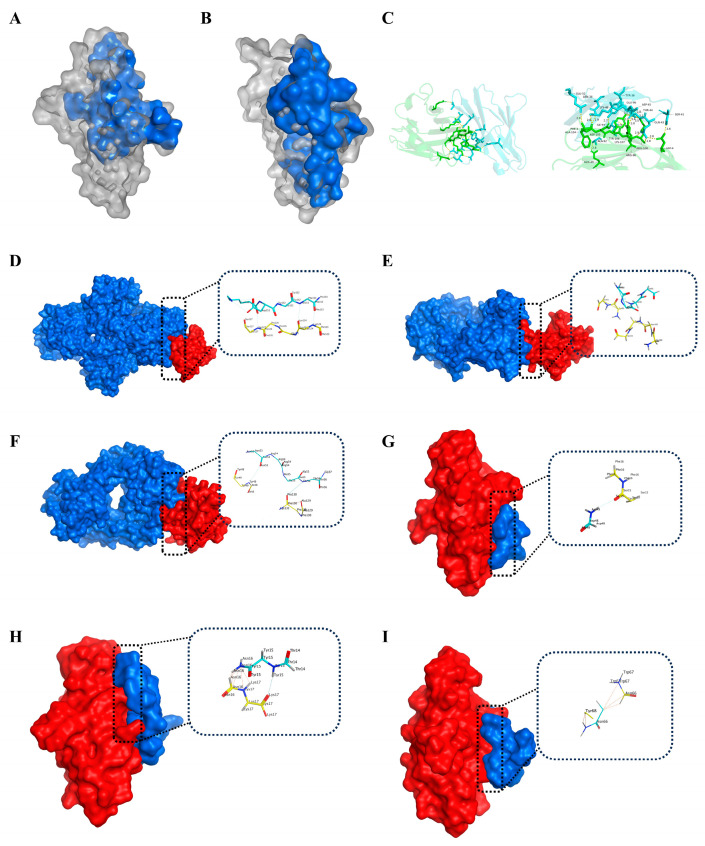
Visualization of the interaction between PD-1 and a variety of bioactive molecules. The active binding sites of (**A**) PD-1 and (**B**) PD-L1 are shown as space-filling models, and the hydrophobic surface is colored in blue. (**C**) The docking result of PD-1 with PD-L1 is shown as a ribbon model, and residues involved in the interaction are shown as a stick model. A variety of macromolecules are complexed with PD-1 and shown as surface models, including (**D**) Pembrolizumab, (**E**) Nivolumab, (**F**) Tislelizumab, (**G**) AUNP-12, (**H**) TPP-1, and (**I**) DPPA-1. PD-1 protein is colored in red, whereas the binding molecules are colored in blue. The stick model is used to illustrate the residues involved in the interaction, which are expanded in the insert panel.

Molecular docking analysis revealed that the PD-1/PD-L1 interface is relatively flat and hydrophobic, with a contact surface area of 1970 Å^2^ (Figure 1A,B). Key interface residues were identified (Figure 1C), consistent with those involved in binding to monoclonal antibodies (Pembrolizumab, Nivolumab, and Tislelizumab; Figure 1D–F, Table S1). Similarly, docking of PD-1 with three reported inhibitory peptides (AUNP-12, TPP-1, and DPPA-1) confirmed overlap with the PD-L1 binding site (Figure 1G–I, Table S2). However, it is important to note that these peptides are linear or branched and thus differ significantly from PD-1-0520, which is designed as a rationally optimized cyclic peptide. The structural differences between these peptide inhibitors, including their lack of intramolecular cyclization, make direct comparisons regarding docking scores, IC_50_ values, and in vivo tumor inhibition challenging.

Nevertheless, the shared contact residues identified in the docking analysis were considered hotspot residues and guided the subsequent rational design of novel cyclic peptides (Table 1). This strategy highlights the potential of PD-1-0520 as a cyclic peptide inhibitor with unique structural and functional advantages, which may offer enhanced binding affinity and tumor inhibition.

### 2.2. Structure-Based Design and Virtual Screening of Cyclic Peptides

**Figure 2:**
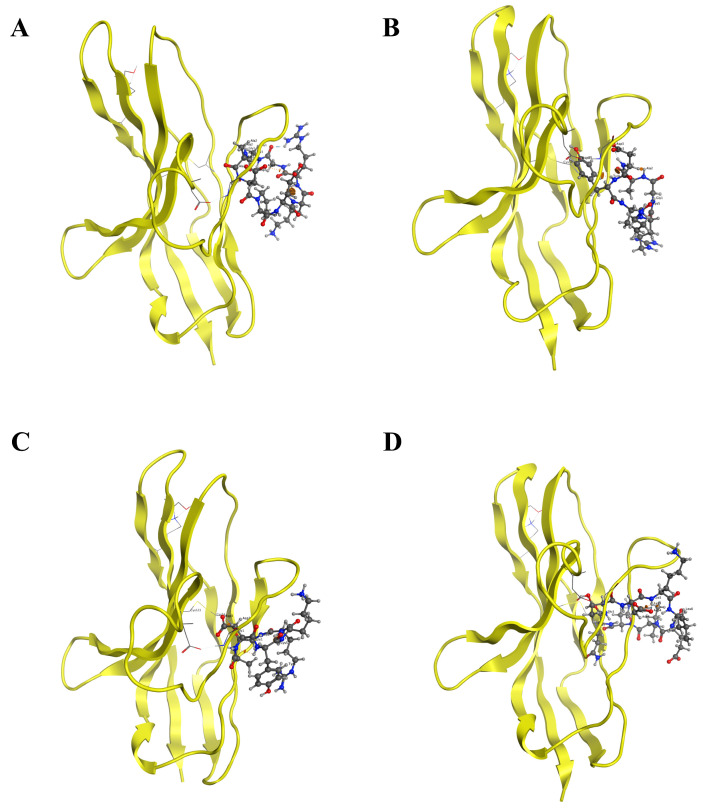
Molecular docking investigation of the interaction between designed peptides and the human PD-1 protein using the HPEPDOCK server. Separate interactions between PD-1 and four peptides were modeled, including PD-1-0514 (**A**), PD-1-0518 (**B**), PD-1-0520 (**C**), and PD-1-0519 (**D**). Peptides were represented as sticks with the atoms colored as carbon gray, nitrogen blue, and oxygen red. The hydrogen-bond interactions with protein residues were represented by black dotted lines.

Five PD-L1 mimetic peptides were docked with PD-1 and PD-L1 using the HPEPDOCK server. All of them exhibited binding to PD-1, with patterns mimicking PD-L1 (Tables S3 and S4, Figure S1). The docking results showed that these peptides were able to bind PD-1 at the canonical PD-L1-interaction surface (Figure S1A), whereas none of them exhibited effective binding at the active site of PD-L1 (Figure S1B). Among them, cyclic peptide BPL1 exhibited the strongest binding affinity. Notably, BPL1 formed specific hydrogen bonds with PD-1 residues Met115 and Glu58 (Figure S1C), and competitive docking confirmed its ability to displace PD-L1 (Figure S1D). Guided by these findings, we designed 22 novel cyclic peptides by substituting hotspot residues in the BPL1 scaffold (Table 2). Docking analysis identified four top candidates, PD-1-0518, PD-1-0519, PD-1-0520, and PD-1-0521, with superior binding scores and hydrogen bond profiles (Figure 2).

From a molecular dynamics perspective, the broad range of docking scores observed for the 22 cyclic peptides (−112 to −287) can be rationalized by differences in cyclization mode, residue substitution, and amino acid conformations. Although all peptides formed at least one hydrogen bond with PD-1, variations in ring size and the position of the cyclization linkage led to distinct degrees of backbone constraint. Peptides with more favorable cyclization geometry and pre-organized backbone conformations were better able to adopt a PD-L1-like binding orientation at the PD-1 interface, thereby strengthening the binding energy. In contrast, peptides containing more flexible loops showed bigger conformational sets and less optimal packing at the interface.

In addition, the strength of the hydrogen-bond and hydrophobic interaction networks played a key role in shaping the docking outcome. High-scoring peptides established multiple hydrogen bonds with PD-1 hotspot residues and engaged a larger hydrophobic surface area, forming a tighter interaction. Peptides with fewer or more transient contacts displayed less stable complexes. Finally, conformational stability also contributed to the predicted activities: cyclic peptides that maintained a compact, low-flexibility conformation during sampling tended to yield more consistent, low-energy poses, whereas structurally flexible peptides produced heterogeneous docking conformations and poorer scores. These MD-related structural features collectively explain why only four candidates particularly PD-1-0520 display superior binding profiles as shown in Table 2.

### 2.3. Molecular Dynamics and Free Energy Analysis

**Figure 3:**
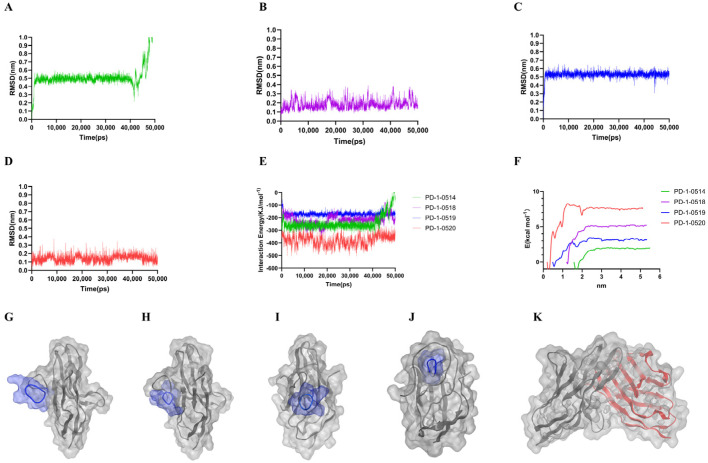
Dynamic simulation results. (**A**–**E**) RMSD analysis of different complexes. (**F**) Binding free energy: Comparison of various systems. (**G**–**K**) The binding modes of peptides and PD-1 with PD-L1 during a 100 ns molecular dynamics simulation.

All-atom molecular dynamics (MD) simulations (5 µs) confirmed the structural stability of the cyclic peptide/PD-1 complexes (Figure 3A–D). Binding free energies (calculated via MM/PBSA) were −48.4, −63.3, −83.7, and −167.4 kJ/mol for PD-1-0514, PD-1-0518, PD-1-0519, and PD-1-0520, respectively (Table 3), indicating enhanced affinity with increasing peptide length. The PD-1-0520 complex demonstrated the most favorable binding, driven primarily by van der Waals interactions, while PD-1-0518 displayed additional electrostatic contributions.

### 2.4. PD-1-0520 Blocks PD-1/PD-L1 Interaction In Vitro

**Figure 4:**
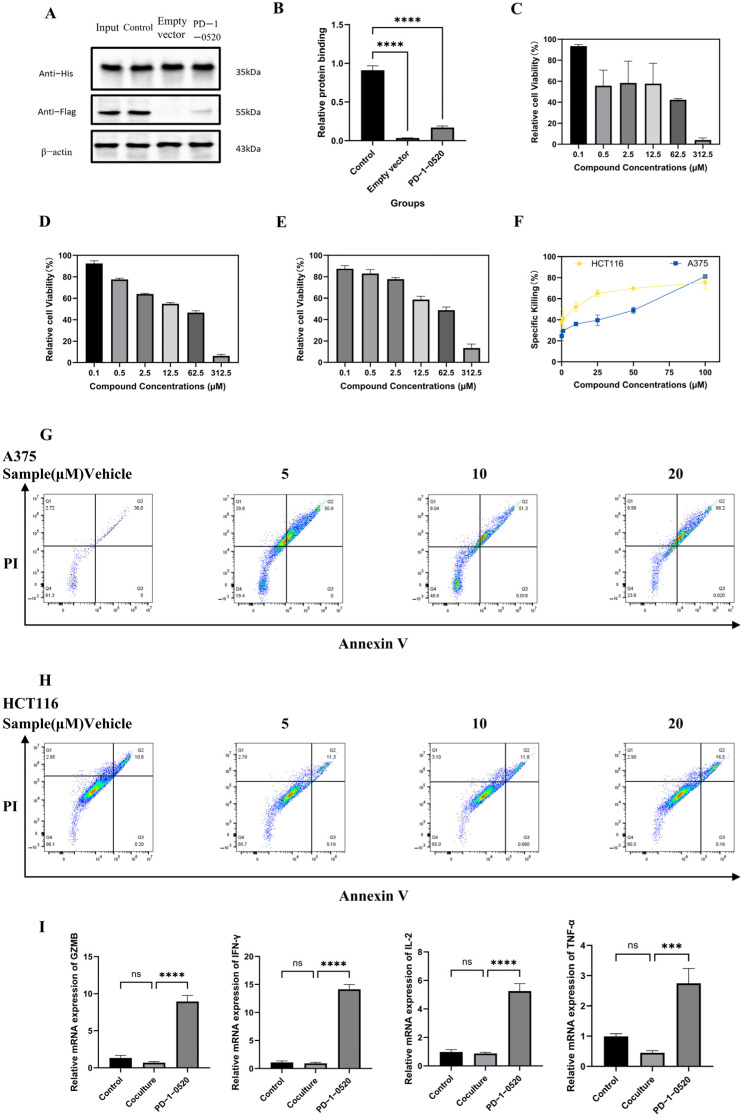
In vitro characterization of the compound in multiple cell lines. (**A**) Pull-down assay showing the binding of His-tagged PD-1 to Flag-tagged PD-L1. His-PD-1 protein was immobilized on Ni-NTA magnetic beads and incubated under four conditions: Input: PD-1 and PD-L1 proteins mixed without beads (loading control). Control: PD-1 + PD-L1 incubated with beads to measure natural binding. Empty vector: Flag-empty protein incubated with beads to assess nonspecific binding.PD-1-0520: PD-1 + PD-L1 incubated with beads in the presence of 10 μM PD-1-0520 to evaluate inhibition of PD-1/PD-L1 interaction. Proteins were detected by Western blot using anti-His (PD-1), anti-Flag (PD-L1), and β-actin antibodies. (**B**) Densitometric quantification of PD-L1 pulled down in the three bead-dependent groups (Control, Empty vector, Inhibitor). Values were normalized to the Control group and represent relative protein binding. (**C**–**E**) Dose–response curves showing relative cell viability of A375, HCT116, and Jurkat cell lines after treatment with varying concentrations of the compound. (**F**) Specific killing (%) of HCT116 and A375 cells by ConA-activated Jurkat T cells at increasing concentrations of PD-1-0520. Killing was calculated relative to tumor-only control wells (0% killing). (**G**,**H**) Annexin V–FITC/PI apoptosis analysis in A375 (**G**) and HCT116 (**H**) cells. (**I**) mRNA expression of GZMB, IFN-γ, IL-2, and TNF-α in cells under different treatment conditions. Includes the following three groups: Control: ConA-stimulated Jurkat T cells cultured alone (baseline activated condition); Coculture: ConA-stimulated Jurkat T cells cocultured with tumor cells at an E:T ratio of 10:1, representing tumor-induced immunosuppression; PD-1-0520: ConA-stimulated Jurkat–tumor coculture treated with PD-1-0520. (*** *p* < 0.001, **** *p* < 0.0001).

A pull-down assay confirmed that PD-1-0520 inhibits PD-1/PD-L1 binding. At 10 µM, PD-L1 binding was reduced to ~20% of control levels (*p* < 0.01, Figure 4A,B), supporting its ability to disrupt immune checkpoint engagement.

### 2.5. In Vitro Antitumor Activity of PD-1-0520

**Figure 5:**
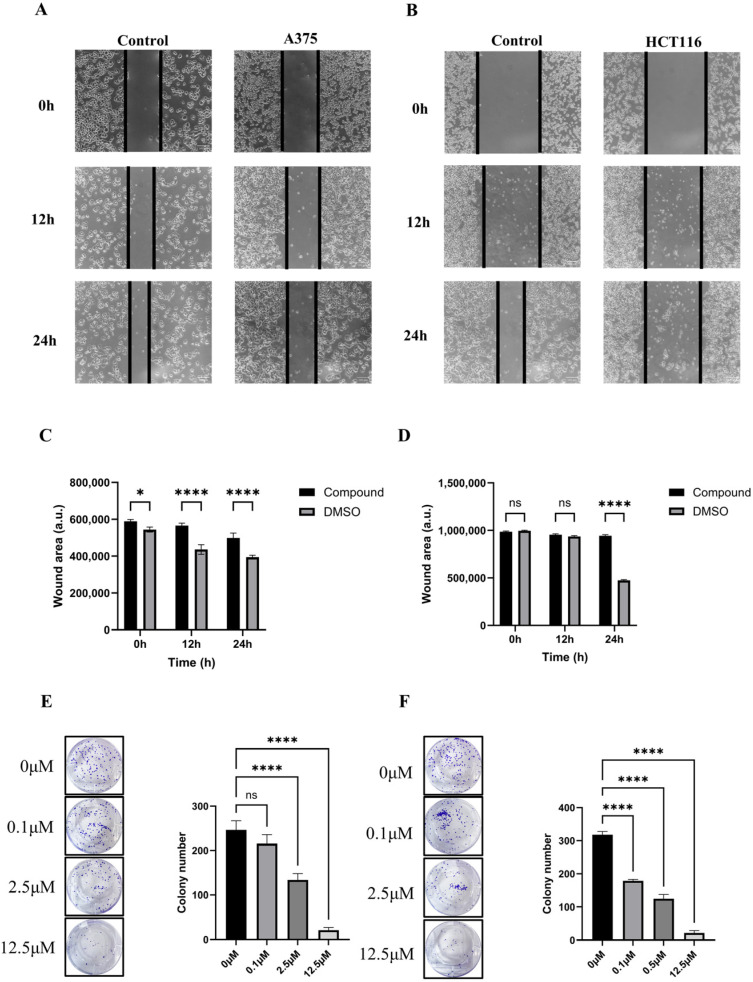
Wound healing and colony formation assays. (**A**,**B**) Representative images from wound healing assays in A375 (**A**) and HCT116 (**B**) cells treated with PD-1-0520 or DMSO at 0, 12, and 24 h. (**C**,**D**) Quantification of wound area over time in A375 (**C**) and HCT116 (**D**) cells measured using ImageJ. Treated groups show significantly slower wound closure in A375 cells at both time points and in HCT116 cells at 24 h, indicating inhibited migration. A few isolated cells are visible in the wound region of treated HCT116 wells at 12–24 h; however, these did not alter the overall wound-area measurements used for quantification. (**E**,**F**) Colony formation assays in A375 (**E**) and HCT116 (**F**) cells treated with different concentrations of PD-1-0520, showing dose-dependent suppression of colony formation. Statistical significance is indicated by **** and ns.Statistical significance: ns = not significant; * *p* < 0.05; **** *p* < 0.0001.

PD-1-0520 demonstrated concentration-dependent cytotoxicity against A375, HCT116, and Jurkat T cells, with IC_50_ values of 17.3 µM, 14.3 µM, and 25.9 µM, respectively (Figure 4C–E). The peptide enhanced T-cell-mediated killing of tumor cells in co-culture (Figure 4F) and significantly increased apoptotic rates, particularly in A375 cells (Figure 4G,H). Furthermore, PD-1-0520 restored immune cytokine expression (GZMB, IFN-γ, IL-2, TNF-α) in Jurkat T cells under tumor-induced immunosuppression (Figure 4I).Scratch assays showed that PD-1-0520 treatment reduced tumor cell migration in both A375 and HCT116 cells, as indicated by the persistently wider wound area in treated groups compared with DMSO controls at 12 and 24 h (Figure 5A–D). Quantitative ImageJ analysis confirmed that PD-1-0520 significantly slowed wound closure in A375 cells at both time points (**** *p* < 0.0001) and in HCT116 cells at 24 h (**** *p* < 0.0001), despite the presence of a few isolated cells within the scratch region in the treated HCT116 group, which did not affect overall wound-area measurements. Clonogenic assays further demonstrated a dose-dependent reduction in colony-forming ability following PD-1-0520 treatment (Figure 5E,F).

### 2.6. In Vivo Antitumor Activity and Immune Activation

**Figure 6:**
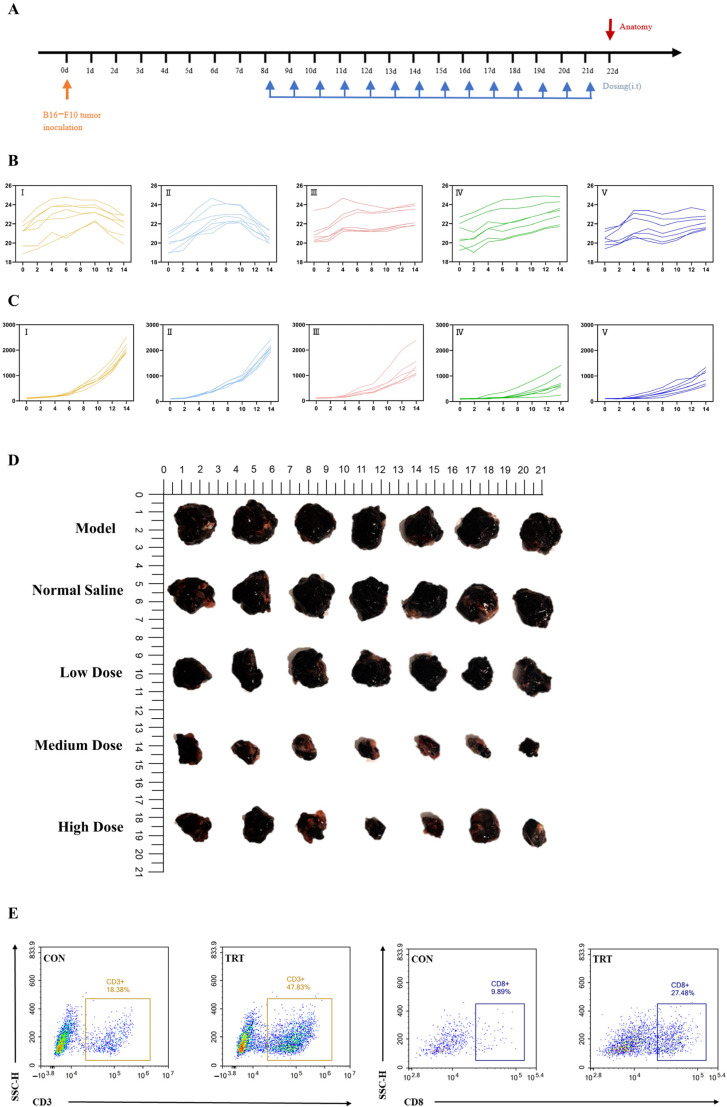
Tumor growth inhibition and immune response in vivo. (**A**) Experimental timeline for B16-F10 tumor inoculation and dosing schedule.(**B**) Body weight changes in the five groups: I = CON, II = NS, III = Low, IV = Medium, V = High. The x-axis shows time in days, and the y-axis shows body weight (g). (**C**) Tumor volume curves for the same five groups: I = CON, II = NS, III = Low, IV = Medium, V = High. The x-axis represents time (days), and the y-axis represents tumor volume (mm^3^). (**D**) Representative images of tumors from different treatment groups. (**E**) Flow cytometric evaluation of CD3^+^ and CD8^+^ immune cell subsets in the control (CON) group and in the medium-dose treatment group (TRT).

**Figure 7:**
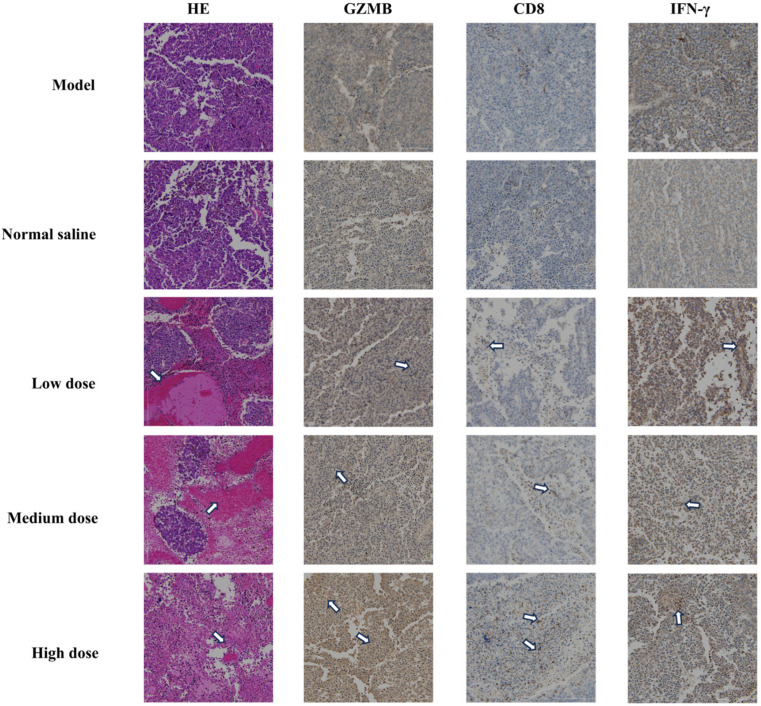
Representative images of tumor sections from the model groups stained with hematoxylin and eosin (HE) and immunohistochemistry for GZMB, CD8, and IFN-γ. Tumor samples were collected from animals treated with normal saline, low dose, medium dose, and high dose of the compound. Left column (HE) shows the tissue morphology, middle columns (GZMB, CD8) highlight immune cell infiltration, and right column (IFN-γ) shows the presence of activated immune responses. Tumor sections from the high-dose group demonstrate increased immune cell infiltration and inflammatory markers compared to the other treatment groups.

**Figure 8:**
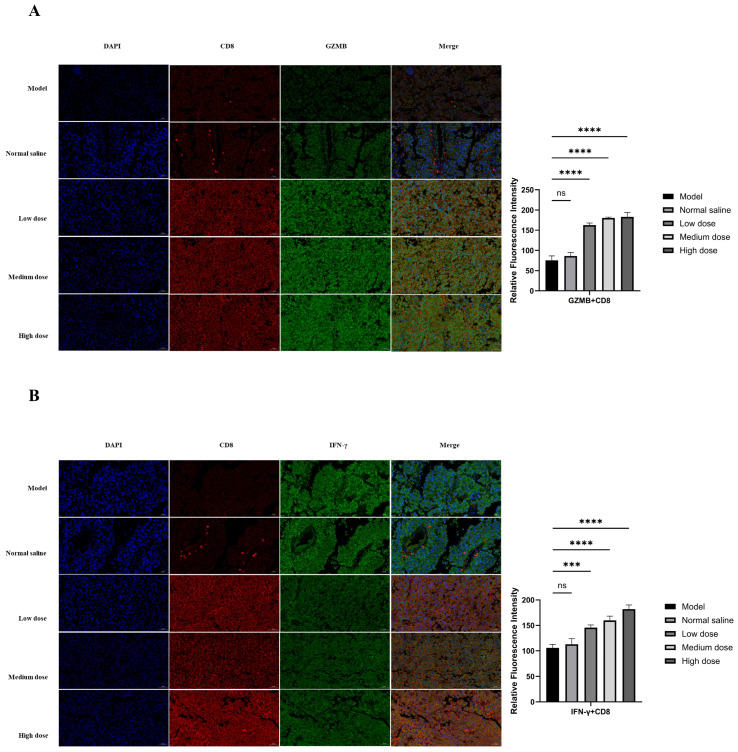
Immunofluorescence analysis of immune cell markers. (**A**) Co-localization of CD8 (red) and GZMB (green) in tumor sections with quantification of GZMB+CD8+ cells. (**B**) Co-localization of CD8 (red) and IFN-γ (green) in tumor sections with quantification of IFN-γ+CD8+ cells. Statistical significance indicated by ****, ***, and ns.

The in vivo dosing schedule is shown in Figure 6A. B16-F10 melanoma cells were inoculated on day 0, followed by intratumoral administration of PD-1-0520 from day 8 to day 21, and tumor tissues were collected on day 22 for subsequent analyses. In this model, PD-1-0520 treatment (5–20 mg/kg) significantly suppressed tumor growth in a dose-dependent manner without causing body-weight loss (Figure 6B,C). Flow cytometry further demonstrated increased infiltration of CD3^+^ and CD8^+^ T cells (Figure 6E), indicating enhanced recruitment of effector T cells into the tumor microenvironment. Histological analyses confirmed these findings (Figure 7). H&E staining showed reduced tumor burden and extensive necrosis in PD-1-0520–treated groups. Immunohistochemical staining revealed marked increases in CD8^+^ T-cell infiltration and strong upregulation of GZMB and IFN-γ expression, particularly in medium- and high-dose groups, indicating enhanced cytotoxic immune activation. Consistently, dual immunofluorescence staining demonstrated increased numbers of CD8^+^GZMB^+^ and CD8^+^IFN-γ^+^ cells after PD-1-0520 treatment (Figure 8A,B), confirming that PD-1-0520 restores intratumoral cytotoxicity and effector cytokine production in vivo. Quantitative ImageJ analysis of fluorescence intensity revealed a dose-dependent increase in activated CD8^+^ T cells. The average GZMB^+^CD8^+^ fluorescence intensity was 75.41 in the model group and 86.10 in the normal saline group, which increased to 162.53, 180.37, and 182.76 in the low-, medium-, and high-dose PD-1-0520 groups, respectively. Similarly, the IFN-γ^+^CD8^+^ fluorescence intensity increased from 105.83 (model) and 112.88 (normal saline) to 145.59, 159.70, and 181.95 in the low-, medium-, and high-dose groups. These results demonstrate that PD-1-0520 enhances CD8^+^ T-cell activation in a dose-dependent manner.

## 3. Discussion

The development of effective immune checkpoint inhibitors has been a cornerstone of modern cancer immunotherapy, with antibodies targeting the PD-1/PD-L1 axis leading the way in clinical success. However, their limitations—including high manufacturing costs, poor tumor penetration, and immune-related adverse events—have spurred the search for alternative modalities. Our study addresses this gap by presenting a structure-guided design strategy for cyclic peptide inhibitors, culminating in the identification of PD-1-0520 as a potent, multi-functional agent capable of disrupting the PD-1/PD-L1 interaction and restoring anti-tumor immunity [14].

The success of PD-1-0520 stems from its rational design, which capitalizes on critical hotspot residues at the PD-1/PD-L1 interface. Through molecular docking analyses of PD-1 with PD-L1, clinically approved antibodies (pembrolizumab, nivolumab, tislelizumab), and reported peptide inhibitors (AUNP-12, DPPA-1), we identified conserved residues that drive binding specificity (Table 1 and Table S1). These residues, including PD-1’s Ile134, Glu136, and Thr76, and PD-L1’s Tyr123, Arg125, and Asp26, form a network of hydrogen bonds, salt bridges, and hydrophobic interactions that stabilize the complex [15]. By incorporating these hotspots into the cyclic peptide scaffold—replacing non-essential residues in the parent peptide BPL1—we enhanced both binding affinity and specificity [16].

Notably, PD-1-0520’s sequence (G-A-D-Y-K-G) was optimized to maximize these interactions: the tyrosine (Y) residue engages in π-π stacking with PD-1’s Ile134, while aspartic acid (D) and lysine (K) form electrostatic interactions with Glu136 and Gln75, respectively (Figure 2C). This design aligns with molecular dynamics simulations, which showed a binding free energy of −167.4 kJ/mol for PD-1-0520—significantly lower than other candidates (Table 3)—driven by strong van der Waals forces and hydrogen bonds (≥11, Table 2) [17]. Such stability ensures prolonged occupancy of the PD-1 binding pocket, effectively blocking PD-L1 engagement, as confirmed by the pull-down assay (Figure 4A,B).

Several peptide-based PD-1 or PD-L1 inhibitors have been reported in the literature, including AUNP-12, TPP-1, and DPPA-1. AUNP-12 is a branched PD-1–derived peptide with low-nanomolar EC_50_ values in mouse splenocytes and human PBMCs, and it exhibits antitumor activity in a Renca renal carcinoma model. TPP-1 is a linear PD-L1-binding peptide with a reported affinity of approximately 95 nM and reduces tumor growth by about 56% in H460 xenografts. DPPA-1, a hydrolysis-resistant D-peptide targeting PD-L1, shows moderate affinity and measurable but relatively modest antitumor efficacy in CT26 colorectal cancer models. In comparison, PD-1-0520 exhibited micromolar IC_50_ values in A375 and HCT116 cells and achieved 68% tumor inhibition at 10 mg/kg in vivo, suggesting that its biological performance is competitive within the landscape of peptide-based immune checkpoint inhibitors.

PD-1-0520 exhibits a unique dual mode of action that distinguishes it from traditional immune checkpoint inhibitors. In vitro, it demonstrated concentration-dependent cytotoxicity against A375 (IC_50_ = 17.3 µM) and HCT116 (IC_50_ = 14.3 µM) tumor cells, with lower toxicity toward Jurkat T cells (IC_50_ = 25.9 µM) (Figure 4C–E). This selectivity suggests direct tumor cell killing, potentially via disruption of intracellular signaling pathways or membrane integrity—though further studies are needed to elucidate this mechanism.

Concurrently, PD-1-0520 reactivates T cell-mediated immunity, a hallmark of effective checkpoint blockade. In co-culture assays, it enhanced T cell-dependent tumor killing (Figure 4F) and restored the expression of key cytokines (GZMB, IFN-γ, IL-2, TNF-α) suppressed by tumor-derived immunosuppression (Figure 4I). These cytokines are critical for effector T cell function: IFN-γ promotes antigen presentation and inhibits tumor angiogenesis, while GZMB mediates direct cytotoxicity against tumor cells [18]. The upregulation of these factors, coupled with increased CD8^+^ T cell infiltration in vivo (Figure 6E), indicates a robust reversal of the immunosuppressive tumor microenvironment.

Cyclic peptides like PD-1-0520 address key limitations of monoclonal antibodies. Their small molecular weight (~600 Da for PD-1-0520) enables deeper tumor penetration compared to antibodies (~150 kDa), which are often restricted to perivascular regions [19]. This is supported by our in vivo data showing uniform distribution of immune activation markers (CD8, GZMB) throughout tumor sections in high-dose PD-1-0520-treated mice (Figure 7), whereas antibody therapies frequently exhibit heterogeneous efficacy in solid tumors.

Additionally, peptide synthesis via solid-phase methods (e.g., Fmoc chemistry) is simpler and more cost-effective than antibody production, which requires mammalian cell culture [20]. This could reduce treatment costs and improve accessibility, particularly in resource-limited settings. PD-1-0520 also showed no systemic toxicity in mice—no weight loss or hematological abnormalities were observed (Figure 6B)—a critical advantage over antibodies, which can induce severe immune-related adverse events (e.g., colitis, pneumonitis) due to off-target immune activation.

The B16-F10 mouse model provided valuable insights into PD-1-0520’s in vivo activity. The 68% tumor inhibition rate in the medium-dose group (10 mg/kg) exceeded that of the high-dose group (20 mg/kg), suggesting a bell-shaped dose–response curve (Table S5) [21]. This may reflect receptor saturation at higher doses or increased clearance, highlighting the importance of optimizing dosing regimens. Immunofluorescence analyses revealed co-localization of CD8^+^ T cells with GZMB and IFN-γ (Figure 8), confirming that infiltrating T cells are functionally activated—a key determinant of anti-tumor efficacy.

Histological analyses further supported immune remodeling: tumor sections from PD-1-0520-treated mice showed increased necrosis (HE staining) and dense infiltration of CD8^+^ T cells, alongside elevated GZMB and IFN-γ (Figure 7). These changes are consistent with a “hot” tumor microenvironment, where active immune responses drive tumor regression. Notably, the absence of systemic toxicity in mice suggests that PD-1-0520 selectively modulates the tumor microenvironment without disrupting peripheral immune homeostasis—an advantage over antibodies that globally activate the immune system.

Our study demonstrates the power of combining computational modeling with experimental validation. Molecular docking (HPEPDOCK) and 100 ns molecular dynamics simulations (GROMACS) enabled the prioritization of candidates with favorable binding kinetics, reducing the need for extensive screening [22]. The correlation between predicted binding free energies (Table 3) and in vitro activity (e.g., IC_50_ values) validates this approach as a cost-effective tool for peptide design.

Notably, the use of multiple orthogonal assays—pull-down (protein–protein interaction), CCK-8 (cytotoxicity), flow cytometry (apoptosis), and cytokine profiling—strengthens the robustness of our findings. Each assay addresses a distinct aspect of PD-1-0520’s activity, from target engagement to functional immune reactivation, providing a comprehensive understanding of its mechanism.

Despite these strengths, our study has limitations. First, the in vivo efficacy was tested only in B16-F10 melanoma; further validation in other syngeneic models (e.g., MC38 colon cancer, Lewis lung carcinoma) is needed to confirm broad applicability [23]. Second, the pharmacokinetic properties of PD-1-0520—including half-life, biodistribution, and metabolism—remain uncharacterized. Cyclic peptides can be prone to proteolytic degradation, so chemical modifications (e.g., N-methylation, PEGylation) may be required to enhance stability.

Future studies should also explore combination therapies. PD-1-0520 could synergize with chemotherapy (e.g., doxorubicin) or radiotherapy, which induce immunogenic cell death, or with other checkpoint inhibitors (e.g., anti-CTLA-4) to further enhance T cell activation [24]. Additionally, structural studies (e.g., X-ray crystallography of the PD-1-0520 complex) would provide atomic-level insights into binding, guiding further optimization [25].

Although our study demonstrates that PD-1-0520 enhances CD8^+^ T-cell infiltration and activation, we did not evaluate additional immune cell subsets such as regulatory T cells, macrophages, or myeloid-derived suppressor cells. A more comprehensive immunophenotyping analysis would provide deeper insights into how PD-1-0520 reshapes the overall tumor microenvironment. Future studies will address this limitation by including a broader panel of immune markers.

Beyond PD-1/PD-L1, our structure-based design strategy offers a blueprint for targeting other protein–protein interactions (PPIs), which are historically challenging drug targets due to their large, flat interfaces. By focusing on hotspot residues and optimizing cyclic peptide scaffolds for stability and affinity, we demonstrate that PPIs can be effectively disrupted with small molecules. This approach could be applied to other immune checkpoints (e.g., CTLA-4/B7, LAG-3/MHC-II) or oncogenic PPIs (e.g., p53/MDM2), expanding the repertoire of targeted therapies [22].

In conclusion, PD-1-0520 represents a promising new class of cyclic peptide immune checkpoint inhibitors, with dual mechanisms of direct cytotoxicity and immune reactivation. Its favorable safety profile, cost-effective synthesis, and potent in vivo activity position it as a viable alternative to antibody-based therapies [26]. Our study not only advances a candidate for clinical development but also validates a rational design pipeline for PPI-targeted peptides, opening new avenues in cancer immunotherapy and beyond.

## 4. Materials and Methods

### 4.1. Experimental Materials

B16-F10, HCT116, and A375 cell lines were purchased from the Cell Bank of the Chinese Academy of Sciences (Shanghai, China); the Jurkat cell line was purchased from the Cell Bank of Wuhan University. B16-F10, HCT116 cells were cultured in DMEM medium containing 10% fetal bovine serum (Hyclone, Logan, UT, USA), 100 units/mL of penicillin, and 100 units/mL of streptomycin in DMEM medium; Jurkat and A375 cells were cultured in RPMI 1640 medium containing the same serum and antibiotic concentrations. All cells were cultured at 37 °C in a 5% CO_2_ humidified environment. The cyclic peptide compounds were all synthesized by Qiangyao Bio (Shanghai, China) with >95% purity. The synthesis was accomplished by an automated peptide synthesizer (AAPPTec Focus XC, Louisville, KY, USA) using the standard Fmoc solid-phase peptide synthesis method with DIC/Cl-HOBt coupling reagent. The crude peptide was purified by cold ether precipitation and washed three times with ether, and finally refined by reversed-phase high-performance liquid chromatography (RP-HPLC), and the mass spectrometry confirmed the structural correctness of the product.

### 4.2. Molecular Docking-Based Hotspot Residue Prediction

Human PD-1/PD-L1 composite crystal structure (PDB ID: 4ZQK) and the composite structures of three marketed PD-1 monoclonal antibodies were obtained from the PDB database: pembrolizumab (PDB ID: 5X8L), Nivolumab (PDB ID: 5X8M), Tislelizumab (PDB ID: 5GRJ). The crystal structures were pre-processed using MOE 2019 software, including removal of solvent molecules, protonation treatment, and energy minimization, and the structures of PD-1, PD-L1 single chain, and monoclonal antibody were extracted and saved as PDB files, respectively. To systematically resolve the key hotspot residues in the PD-1 binding pocket, the HDOCK server (http://hdock.phys.hust.edu.cn/, accessed on 15 March 2024) was used for protein–protein docking analysis of PD-1 with PD-L1 and monoclonal antibodies [27]. Meanwhile, three reported PD-1 peptide inhibitors (AUNP-12, TPP-1 and DPPA-1) were collected for peptide-protein docking studies using the HPEPDOCK server (http://huanglab.phys.hust.edu.cn/hpepdock/, accessed on 15 March 2024). The key hotspot residue information was summarized by comprehensively analyzing the above docking results (Table 1). All molecular docking results were analyzed using PyMOL 3.0 (Schrödinger, LLC, New York, NY, USA) for 3D visualization [28].

### 4.3. Cyclic Peptide Design Strategy

Before designing the cyclic peptide library, five PD-L1 mimetic peptides previously generated in our laboratory through in silico modeling of the PD-1/PD-L1 interface were used as the initial virtual scaffolds for screening. The sequences of these computational mimetic peptides are provided in Supplementary Tables S3 and S4. Based on the results of hotspot residue analysis, we designed 22 cyclic peptide candidate molecules (see Table 2 for sequence information). The design strategy takes the sequence and secondary structure of the template cyclic peptide BPL1 as a starting point, and selectively replaces its amino acids interacting with the PD-1 active region with the hotspot residues identified above [29]. Considering the hydrophobic character of the PD-1 active site, hydrophobic residues such as glycine, alanine, and phenylalanine were preferred. Polar amino acids capable of forming hydrogen bonds with the PD-1 active site were also introduced to enhance binding specificity. Molecular docking of 22 designed cyclic peptides with PD-1 proteins was performed using the HPEPDOCK server, and the molecules were sorted and screened based on docking scores and the number of hydrogen bonds. Cyclic peptides with docking score < −210 and hydrogen bond number ≥ 5 were selected as potential PD-1 inhibitor candidate molecules to ensure sufficient binding affinity and stability.

### 4.4. Molecular Dynamics Simulations and Binding Free Energy Calculations

Highly ranked peptide–protein complexes were selected as initial coordinates for all-atom molecular dynamics simulations using the GROMACS 2022.2 package (GNU General Public License; http://www.gromacs.org, accessed on 18 April 2024) in combination with the Amber99SB-ILDN force field. Each simulated system contained a cyclic peptide molecule and a PD-1 protein, which were solvated using the TIP3P water molecule model and the system charge neutralized with Cl^−^ or Na^+^ ions. Solvation of the box required a minimum distance of 1.2 nm between the cyclic peptide and the box boundary. The simulation process was divided into multiple phases: first, energy minimization was performed using the most rapid descent method; followed by NVT system equilibrium at 100 ps and NPT system equilibrium at 1 ns, during which weakly harmonic positional constraints were imposed on the heavy atoms. The NVT system was temperature-controlled using a Berendsen thermal bath, and the NPT system was equilibrated at 1 atm at 310 K using a Parrinello-Rahman pressure coupler. The production phase was subjected to a 100 ns MD simulation with an integration step of 2 fs and trajectories recorded every 10 ps [27]. Covalent bonds involving hydrogen atoms were constrained using the LINCS algorithm, and long-range electrostatic interactions were handled by the particle mesh Ewald method with a real-space cutoff distance of 10 Å. Trajectory visualization and analysis were performed using Visual Molecular Dynamics (VMD) software, and statistical analysis was performed by the GROMACS built-in tool. The binding free energy between the cyclic peptide and PD-1 protein was finally calculated using the Molecular Mechanics/Poisson-Boltzmann surface area (MM-PBSA) method to assess the binding stability.

### 4.5. Protein Interaction Blocking Assay (Pull-Down Assay)

In the protein interaction blocking assay, Ni-NTA magnetic beads were first used to bind His-tagged PD-1 fusion proteins to immobilize the PD-1 protein. Take 20 µL of Ni-NTA magnetic beads into a 1.5 mL EP tube, add 500 µL of PBS buffer, gently mix, and adsorb the beads on a magnetic rack, discard the supernatant, and repeat the wash 2 times. Next, 1–2 µg of His-tagged PD-1 protein was added to make up the volume to 100 µL, and incubated for 1 h at 4 °C with gentle rotation to allow the PD-1 protein to bind to the surface of the magnetic beads. At the end of the incubation, the magnetic beads were washed three times with PBS to remove unbound protein. Subsequently, the magnetic beads were divided into four treatment groups: Input group: PD-1 and PD-L1 proteins were added, but the magnetic beads were not used, and were only used to confirm the expression and binding of both. Control group: 1 µg of Flag-tagged PD-L1 protein was added without inhibitors and was used to assess the natural binding of PD-1 to PD-L1. Empty group: 1 µg of Flag-tagged empty protein without inhibitor was added to exclude binding of magnetic beads to non-specific proteins. Inhibitor group: 1 µg Flag-tagged PD-L1 protein was added with 10 µM PD-1-0520 inhibitor, which was used to assess the inhibitory effect of the inhibitor on PD-1 binding to PD-L1. All groups were incubated at 4 °C for 2 h with gentle rotation. At the end of the incubation, the magnetic beads were washed 3 times with PBS to remove unbound proteins. Then, 20–30 µL of protein upload buffer (containing SDS and reducing agent) was added and heated at 95 °C for 5 min to cleave the magnetic bead-bound proteins. Proteins were separated by SDS-PAGE, transferred to a PVDF membrane, and analyzed by Western Blot using Anti-His antibody (to detect PD-1), Anti-Flag antibody (to detect PD-L1), and β-actin antibody (internal reference). After incubation with HRP-labeled secondary antibody, the binding of PD-1 to PD-L1 and the effect of inhibitors were assessed by ECL luminescence development [30].

### 4.6. Cell Viability Assay

Collect the logarithmic growth phase cells, adjust the concentration to 5 × 10^3^ cells/well inoculate in a 96-well plate. After the cells were attached to the wall, seven different concentrations of compounds were given to treatments, while setting up zero-adjusted wells and control wells, and six parallel wells were set up for each treatment, which were placed in the incubator at 37 °C, 5% CO_2_. After 24, 48, and 72 h, respectively, 10 μL of CCK-8 solution was added to each well, and the optical density values were measured at 450 nm with an enzyme meter after 2 h of incubation at 37 °C [31].

Cell survival = (As − Ab)/(Ac − Ab) × 100%

where As is the optical density of experimental wells, Ac is the optical density of control wells, and Ab is the optical density of blank wells. Based on the cell survival at different drug concentrations, a dose-effect curve was fitted, and the half inhibitory concentration (IC_50_) was calculated using GraphPad Prism 8 software.

### 4.7. T-Cell-Mediated Tumor Cell Killing Assay

Jurkat T cells in the logarithmic growth phase were collected and stimulated with 5 μg/mL of conA (Concanavalin A) for 48 h for preactivation. Tumor cells were inoculated in 96-well plates at a density of 5 × 10^3^ cells per well, and the preactivated Jurkat cells were added after they adhered to the wall. The effector-to-target cell ratios (E:T) were set at 10:1. Different concentrations of the compounds to be tested were added at the same time, and the cells were incubated for 48 h. At the end of the experiment, suspended Jurkat cells were removed by PBS washing, and tumor cell viability was measured using the CCK-8 assay [32]. Tumor-only wells (tumor cells plated at the same density without Jurkat T cells) served as the 0% killing control (Ac). Blank wells contained medium + CCK-8 only (Ab). The killing rate was calculated as:

Relative tumor killing (%) = [1 − (As − Ab)/(Ac − Ab)] × 100%

where As is the absorbance of cocultures.

### 4.8. Detection of Apoptosis by Flow Cytometry

A375 and HCT116 cells were treated with 0, 5, 10, and 20 μM of PD-1-0520 for 48 h, respectively. Cells and culture supernatants were collected, centrifuged at 800 rpm for 3 min, the supernatants were discarded, and the cells were resuspended with PBS, centrifuged at 4 °C, 300× *g* for 5 min, and the supernatants were discarded again. Cells were resuspended with 1× Binding Buffer, and the concentration was adjusted to approximately 5 × 10^6^ cells/mL [33]. 1.5 μL of Annexin V-FITC and 1.5 μL of propidium iodide (PI) were added, and the cells were mixed and incubated for 30 min away from light. Subsequently, 350 μL Binding Buffer was added to resuspend the cells, and apoptosis was detected using flow cytometry within 1 h. Early apoptosis (Annexin V^+^/PI^−^) and late apoptosis (Annexin V^+^/PI^+^) cell proportions.

### 4.9. Cell Scratch Assay

A375 and HCT116 cells were digested with 0.25% trypsin for 2 min, collected by centrifugation at 1000 rpm for 5 min, and resuspended in complete medium. The cell density was adjusted to 5 × 10^5^ cells/well for each condition and seeded into 6-well plates. Each well was then supplemented with 2 mL of medium containing 20% fetal bovine serum (FBS). Cells were cultured at 37 °C with 5% CO_2_ until the monolayer reached approximately 90% confluence. Scratches were made using a sterile 200 μL pipette tip, and reference lines (0.5–1 cm apart) were drawn on the back of the culture plate to ensure the consistency of scratch width and position across all wells. After scratching, the wells were gently washed three times with PBS to remove detached cells. Next, serum-free medium and 10µM PD-1-0520 were added to the corresponding wells. To simulate an immune co-culture system, Jurkat T cells were pre-activated by incubation with 5 μg/mL Con-A (E:T ratio of 10:1), and then added to each well [34]. The cultures were maintained at 37 °C with 5% CO_2_, and the healing of the scratches was recorded by photographing at fixed positions at 0, 12, and 24 h. The cell migration distance was quantified using ImageJ 1.54 g (National Institutes of Health, Bethesda, MD, USA) by measuring the wound area at each time point.

### 4.10. Cell Clone Formation Assay

A375 and HCT116 cells in logarithmic growth phase were taken, digested with 0.25% trypsin and blown into single-cell suspensions, counted and diluted to 500 cells per well, and inoculated into 6-well plates. DMEM medium containing 20% fetal bovine serum was added to each well and incubated at 37 °C, 5% CO_2_ for 1–2 weeks until clones were formed to the naked eye. The drug treatment group was equipped with four concentrations: 0 µM, 0.1 µM, 2.5 µM, and 12.5 µM. The drug concentration of each group was added to the medium for different concentrations of drug treatment [35]. After the clones were formed, the culture was terminated, the cells were washed twice with PBS, fixed using 4% paraformaldehyde for 15 min, dried naturally, and then stained with 0.1% crystal violet for 10 min to remove the excess dye and washed with deionized water. After drying, the number of clones in each well was photographed and counted under a microscope.

### 4.11. Real-Time PCR to Detect Cytokine Expression

Jurkat T cells were first stimulated with ConA and then cultured either alone (ConA-stimulated Jurkat control) or cocultured with tumor cells at an effector-to-target (E:T) ratio of 10:1 for 48 h. In the coculture system, PD-1-0520 (10 μM) was added to assess its ability to reverse tumor-induced immunosuppression. After 48 h, Jurkat cells were collected from the supernatant for RNA extraction. Total RNA was isolated using Trizol reagent according to the manufacturer’s protocol and reverse-transcribed into cDNA using the SPARKscript II RT Plus kit. mRNA levels of GZMB, IFN-γ, IL-2, and TNF-α were quantified using SYBR Green–based real-time PCR, with β-actin as the internal control. Relative expression (RQ, 2^−ΔΔCt^) was calculated using Ct values. Primer sequences are listed in Table 4.

### 4.12. Animal Experiments and Tissue Analysis

In this experiment, 6–8-week-old male SPF-grade C57BL/6 mice were purchased from Guangzhou Experimental Animal Center. 1 × 10^6^ B16-F10 cells were suspended in saline and injected subcutaneously into the root of the right thigh of mice. When the tumor volume reached 100 mm^3^, intraperitoneal administration of 0.1 mL/10 g body weight was started once daily for 14 consecutive days. The drug treatment fractions were high-dose group (20 mg/kg), medium-dose group (10 mg/kg), and low-dose group (5 mg/kg), and the control group was given an equal volume of solvent (DMSO:PEG300:Tween-80:saline = 10%:40%:5%:45%). The model group refers to tumor-bearing mice that did not receive any treatment or saline injections. These mice were used as a baseline to assess the effects of PD-1-0520 and other treatments. Mouse body weight and tumor volume were monitored daily during the treatment period (calculation formula: length × width^2^ × 0.5). On the 2nd day after the end of treatment, orbital blood was collected to determine blood counts. Subsequently, tumor tissues were dissected and removed, fixed using 4% paraformaldehyde, paraffin-embedded, and sectioned at a thickness of 6 μm for HE staining and immunohistochemical (IHC) analysis. In IHC experiments, the sections were first blocked with 20% mouse serum, then incubated with rabbit anti-GZMB, CD8, and IFN-γ primary antibody (Servicebio, Wuhan, China) overnight at 4 °C, followed by incubation with HRP-labeled secondary antibody, and finally stained with DAPI for 10 min, washed with PBS, and observed under a microscope (Olympus CX33,Olympus Corporation, Tokyo, Japan) for observation. In addition, immunofluorescence double staining was used to analyze the co-expression of CD8+ with GZMB and CD8+ with IFN-γ. The antibodies used in this study are listed in Table S6 of the Supplementary Information. This includes details on the antibody application, target, host species, manufacturer, and country of origin.

## Figures and Tables

**Table 1:** The residues involved in the interaction between humanized PD-1 and PD-L1.

PD-1 Residues	PD-L1 Residues	Interaction Types
Ile134, Glu136, Thr76	Tyr123	Alkyl-π hyperconjugation, H-bond
Asn66	Ala121	H-bond
Glu136	Arg125, Arg113	H-bond, salt bridge
Gln75	Arg125, Asp26	H-bond
Ile134	Glu58, Glu60	H-bond
Ala132	Gln66	H-bond
Thr76	Tyr124	H-bond
Lys78	Phe19	H-bond
Asn66	Ala121	H-bond
Asn34	Phe2,Ala204	H-bond
Aer41	Asp9	H-bond

**Table 2:** Docking results of peptides and PD-1 using HPEPDOCK.

Protein	Peptide	Sequence	Scores	Number of Hbonds
PD-1	PD-1-0201	**D-**W**-F-K-A-F-**W	−168.8490856	2
	PD-1-0212	**D-**W**-F-R-A-F-Y**	−153.0645272	2
	PD-1-0305	**D-**W**-F-K-**P**-F-Y**	−165.3617216	1
	PD-1-0310	**D-**W**-Y-K-A-F-Y**	−150.91688	3
	PD-1-0317	**E-**W**-F-K-A-F-Y**	−163.098596	5
	PD-1-0326	**D-**W**-F-K-**G**-F-Y**	−159.6430304	1
	PD-1-0329	**D-**W**-F-K-**C**-F-Y-**C	−168.3935827	2
	PD-1-0402	**D-**W**-F-N-A-F-Y**	−141.9094456	2
	PD-1-0411	**D-**W**-F-K-A-**W**-Y**	−172.3911617	1
	PD-1-0415	**D-**W**-F-K-A-F-**H	−158.3387286	5
	PD-1-0423	**D-**W**-F-K-**S**-F-Y**	−138.1337358	3
	PD-1-0503	G**-**W**-F-K-A-F-Y-**G	−167.2862139	1
	PD-1-0514	G**-A-D-**P**-E-K-R-**G	−211.771068	7
	PD-1-0518	G**-A-D-Y-K-R**	−248.83294	8
	PD-1-0519	**D-A-D-Y-K-R**	−213.1262656	5
	PD-1-0617	**N-Q-T-D-K-**L**-E-I**	−263.3777792	1
	PD-1-0520	G**-A-D-Y-K-**G	−287.7939296	11
	PD-1-0529	G**-A-D-**P**-E-K**	−112.2599597	2
	PD-1-0604	G**-K-D-F-Y-A**	−129.4551328	2
	PD-1-0609	**E-**M**-E-D-F-A**	−157.1481112	2
	PD-1-0619	**D-W-F-K-**P**-A-**G	−132.4389846	1
	PD-1-0623	**D-E-**M**-E-D-**G	−128.5718201	2

Note: Residues shown in bold represent the predicted hot-spot residues that contribute significantly to the peptide–PD-1 interaction.

**Table 3:** The MM/PBSA binding free energy of the complex during the 500 ns MD simulation.

	PD-1-0514	PD-1-0518	PD-1-0519	PD-1-0520	PD-1
Binding (kJ/mol)	−48.382	−63.251	−83.743	−167.351	−90.052
MM (kJ/mol)	−254.604	−327.048	−538.087	−627.593	−983.159
PB (kJ/mol)	203.937	437.715	552.547	654.193	894.752
SA (kJ/mol)	−23.658	−31.926	−40.293	−47.076	−49.219
COU (kJ/mol)	−54.683	−436.614	−157.610	−499.379	−683.425
VDW (kJ/mol)	−221.314	−194.803	−304.674	−298.015	−337.686
PB_com_ (kJ/mol)	−5136.531	−4792.264	−5328.217	−5753.738	−6783.682
PB_pro_ (kJ/mol)	−4284.739	−4592.976	−4736.592	−5231.265	−5064.689
PB_lig_ (kJ/mol)	−1375.384	−1683.242	−2763.218	−3258.629	−4758.405
SA_com_ (kJ/mol)	153.352	167.427	179.969	198.859	283.371
SA_pro_ (kJ/mol)	159.527	158.736	159.037	161.482	163.648
SA_lig_ (kJ/mol)	57.735	68.273	93.304	126.483	175.732
Pre-residue of the peptide (kJ/mol)	−3.233	−5.964	−2.958	−10.114	/

Note: MM indicates the gas-phase molecular mechanics energies, including the van der Waals and electrostatic energies (ΔGvdw + ΔGele); PB indicates the polar solvation free energy (ΔGpolar); SA indicates the non-polar solvation free energy (ΔGnonpolar) that is estimated using the solvent-accessible surface area (SASA); COU is the electrostatic energy (ΔGele); VDW is the van der Waals energy (ΔGvdw). The subscripts “com”, “pro”, and “lig” are the related energies of the complex, PD-1 protein, and individual peptide, respectively.

**Table 4:** Real-time PCR primers.

Primer	Sequence	Number
IL-2 (human)	Forward: GTT GTT TCA GAT CCC TTT AGT TCC A	SEQ ID NO.1
	Reverse: ACA GAA CTG AAA CAT CTT CAG TGT C	SEQ ID NO.2
GZMB (human)	Forward: GCA GGA AGA TCG AAA GTG CG	SEQ ID NO.3
	Reverse: TAC AGC GGG GGC TTA GTT TG	SEQ ID NO.4
TNF-α (human)	Forward: AGC CCA TGT TGT AGC AAA CC	SEQ ID NO.5
	Reverse: GGA AGA CCC CTC CCA GAT AG	SEQ ID NO.6
IFN-γ (human)	Forward: GAA AAG CTG ACT AAT TAT TCG GTA ACT G	SEQ ID NO.7
	Reverse: GTT CAG CCA TCA CTT GGA TGA G	SEQ ID NO.8
Actin-β (human)	Forward: ATT CCT TCA GCA GCA TCC	SEQ ID NO.9
	Reverse: CAA TGC CAG GGT ACA TGG TG	SEQ ID NO.10

## Data Availability

The original contributions presented in this study are included in the article/Supplementary Material. Further inquiries can be directed to the corresponding authors.

## Supplementary Materials

The following supporting information can be downloaded at: https://www.mdpi.com/article/10.3390/ijms262311308/s1.

## Abbreviations

The following abbreviations are used in this manuscript:

PPI	protein–protein interaction
PD-1	programmed death receptor-1
PD-L1	PD-1 ligand 1
mAbs	monoclonal Antibodies
MD	Molecular dynamics
RMSD	root-mean-square deviation
MM-PBSA	molecular mechanism/Poisson–Boltzmann surface area
IC_50_	Half-maximal inhibitory concentration
IFN-γ	interferon γ
IL-2	interleukin 2
TNF-α	Tumor Necrosis Factor-α
GZMB	granzyme B
IHC	immunohistochemistry
ANOVA	analysis of variance
