# Supplementary material for: Structure-Guided Design of Cyclic Peptide: A Potent Inhibitor Targeting PD-1/PD-L1 Axis with Antitumor Activity
Source: Int J Mol Sci. 2025 Nov 22;26(23):11308. doi: 10.3390/ijms262311308 (PMC12692123; doi:10.3390/ijms262311308)
Supplement: Supplementary file 1 [file ijms-26-11308-s001.zip › Table.pdf]

Table S1 Hotspot Residues of Pembrolizumab, Nivolumab, and Tislelizumab Binding to PD-1

	Hot residues
Pembrolizumab	Leu25, Pro89, Ile126, Leu128, Ile134,Arg86, Asp85,Val64, Asn66
Nivolumab	Ser27, Thr28, Asp29,Lys78, Ala79,Pro89, Leu128
Tislelizumab	Pro89, Leu128, Ile134,Asp85, Arg86,Ile126, Val64

Table S2 Sequence of PD-1 peptides inhibitors

Peptide	Sequence	Ref
AUNP-12	SNTSESFKFRVTQLAPKAQIKE	1
TPP-1	SGQYASYHCWCWRDPGRSGGS	2
DPPA-1	NYSKPTDRQYHF	3

Table S3 Sequence Information of Five PD-L1 Mimetic Peptides

Peptide	Sequence
BP1	D-K-V-A-E-K-L
BP2	D-K-A-V-E-Y-F
BP3	D-A-Y-E-F-G-K
BPL1	D-W-F-K-A-F-Y
BPL2	D-W-L-K-A-F-G

Table S4 Docking Score Summary of Five PD-L1 Mimetic Peptides with PD-1/PD-L1

Target Protein	PD-L1 mimetic peptides	Number of H-bonds	Scores
PD-1	BP1	1	-193.3671356
	BP2	2	-183.1092781
	BP3	1	-270.2061433
	BPL1	5	-238.9216201
	BPL2	3	-210.2153714
PD-L1	BP1	1	-93.1744738
	BP2	1	-84.67284312
	BP3	2	-87.19124083
	BPL1	1	-98.53218329
	BPL2	1	-75.10881241

Table S5 The anti-tumor effect of treated groups (mean ± SEM, n = 7)

	Group	Tumor volume(cm^3)	Tumor weight(g)	Tumor inhibition rate(%)
I	Model	2.20±0.31	2.93±0.82	/
II	Normal saline	2.18±0.25	2.87±0.74	/
III	Low dose	1.53±0.85	1.63±0.61	44.36860068
IV	Medium dose	0.83±0.58	0.91±0.56	68.94197952
V	High dose	0.97±0.36	1.13±0.33	61.4334471

Table S6 Antibodies used in this study

**Application**	**Target**	**Host**	**Manufacturer**	**Country**
IHC	CD8	Rabbit	Servicebio	China
IHC	Granzyme B	Rabbit	Servicebio	China
IHC	IFN-γ	Rabbit	Servicebio	China
IF	CD8	Rabbit	Servicebio	China
IF	Granzyme B	Mouse	Servicebio	China
IF	IFN-γ	Rabbit	Servicebio	China
IF Secondary	Goat anti-rabbit IgG	Goat	Servicebio	China
IF Secondary	Goat anti-mouse IgG	Goat	Servicebio	China
Flow cytometry	CD3-FITC	Rabbit	Servicebio	China
Flow cytometry	CD8-PE	Rabbit	Servicebio	China
